# Perspectives of racially minoritized youth with disabilities on addressing ableism and other forms of discrimination

**DOI:** 10.1371/journal.pone.0338179

**Published:** 2025-12-04

**Authors:** Sally Lindsay, Janice Phonepraseuth, Van Slothouber, Jaden Lo, Jennifer N. Stinson, Sharon Smile

**Affiliations:** 1 Bloorview Research Institute, Holland Bloorview Kids Rehabilitation Hospital, Toronto, Ontario, United States of America; 2 Department of Occupational Science & Occupational Therapy, University of Toronto, Toronto, Ontario, United States of America; 3 Rehabilitation Sciences Institute, University of Toronto, Toronto, Ontario, United States of America; 4 Health Sciences, McMaster University, Hamilton, Ontario, United States of America; 5 Research Institute, Hospital for Sick Children, Toronto, Ontario, United States of America; 6 Lawrence S. Bloomberg Faculty of Nursing, University of Toronto, Toronto, Ontario, United States of America; 7 Department of Pediatrics, University of British Columbia, Vancouver, British Columbia, Canada; University of Amsterdam, NETHERLANDS, KINGDOM OF THE

## Abstract

**Background:**

Racially minoritized youth with disabilities often encounter more extensive forms of discrimination. However, little is known about youth perspectives for addressing disability-related and other forms of discrimination, which is important for enhancing the participation and inclusion of youth with disabilities. This study explored the recommendations of youth with disabilities for addressing barriers and multiple forms of discrimination.

**Methods:**

This study involved in-depth interviews with a purposive sample of 20 youth with disabilities. We applied an inductive thematic analysis to the transcripts.

**Results:**

Our findings highlighted the following key themes: (1) addressing barriers in healthcare, education, employment and the legal system; (2) community, social supports and resources; (3) advocacy; and (4) inclusive policies and youth involvement.

**Conclusions:**

There is a critical need for more inclusive services and support for youth with disabilities, especially those with multiple minoritized identities, to foster safe environments and quality of life.

## Introduction

Youth with disabilities often experience stigma and discrimination, social exclusion, and a lack of access to services [1–3]. They encounter barriers and disability-related discrimination, (ableism) across various domains including healthcare, education, employment, and the broader community [4]. Ableism refers to beliefs and practices that marginalize people with disabilities and judges them by able-bodied standards [5]. Forms of ableism include stereotyping, prejudice, discrimination, social exclusion, social oppression and exploitative or abusive conduct towards people with disabilities, which can occur at multiple levels (e.g., individual, organizational, societal) [6]. A recent review exploring the experiences of ableism among children with disabilities reported that this group contends with several forms of ableism at the individual, familial, institutional, and societal levels [2]. Youth with disabilities who identify as belonging to multiple minoritized identities, such as racial and sex/gender minoritized, commonly experience additional and more complex forms of discrimination [4,7,8]. The term minoritized refers to how people in positions of power treat others differently based on their multiple identities (e.g., race, ethnicity, disability, gender, etc.) [9]. For example, a qualitative systematic review focusing on the lived experiences of racially minoritized youth with disabilities found that they encountered challenges accessing and navigating services, stigma, racism, and communication barriers [10]. Other studies show that the type of disability, cultural factors, language barriers, knowledge of available services and resources, and disability awareness affect experiences of discrimination among racially minoritized youth with disabilities [4,11].

Experiences of ableism often results in a fear of disclosing a minoritized identity such as a disability [12,13]. Ableism, especially at a young age, can affect a youth’s physical, psychological, and social well-being and quality of life [10,12,14]. Research indicates that ableism can increase the risk of psychological distress, anxiety, and depression among children and youth with disabilities [15–17]. There is a critical need for further strategies to reduce ableism. Much less research has explored youth suggestions for addressing discrimination, especially from the perspectives of racially minoritized youth with disabilities [18]. Within Canada, where this study was conducted, youth with disabilities comprise approximately 15.4% of those belonging to a racially minoritized group [19]. Additionally, rates of ableism are rising among younger individuals compared to older individuals [20]. Although racial and ethnic inequalities exist for youth with disabilities, health and social service providers often overlook the need to apply a culturally safe approach to service delivery [21,22], which may result in adverse health and social outcomes [10,23]. Of the limited research on this topic, studies show that racially minoritized children with disabilities often experience disability discrimination differently. Specifically, Black and Asian children must also contend with racism and inequitable access to services, compared to white children with disabilities who do not [23]. Similarly, discrimination affects the quality of care that racially minoritized youth with autism receive including racial disparities in diagnosis, healthcare services, care co-ordination, and medications prescribed [4].

### Coping strategies

Research shows that youth with disabilities demonstrate capacity for coping strategies despite encountering an extraordinary amount of ableism and negative attitudes [24]. For example, youth with acquired brain injuries reported coping strategies, such as disclosing their condition to others and learning how to self-manage their symptoms and/or ask for accommodations or supports [10,25]. Further, a study on strategies for improving disability awareness and the social inclusion of mostly white children and youth with cerebral palsy found that disclosing their condition to others, creating awareness about disability, and building a peer support network helped them to cope with bullying and social exclusion [24]. Racially minoritized youth with disabilities displayed coping strategies at school, work, and in the community through seeking out mentors, role models, social supports, and engaging in advocacy [4,26]. A recent review highlighted that Asian youth with disabilities developed self-care strategies and accessed social supports to deal with the various forms of discrimination they experienced [2].

### Theoretical perspective: Intersectionality

We used an intersectional framework to inform our study [27]. Kimberlé Crenshaw [28] originally coined intersectionality [29] to demonstrate how multiple social identities, such as race, disability, gender, and sexual orientation, intersect at the micro level of individual experiences to reveal interlocking systems of privilege and oppression such as racism, ableism, sexism, classism, at the macro socio-structural level [30,31]. Within this theory, researchers view social identities not as independent and unidimensional but as multiple and intersecting [31]. This theoretical approach centers individuals from historically oppressed and marginalized groups, emphasizing how their intersecting social identities at the micro level interact with broader structural forces at the macro level [31]. Intersectionality emphasizes that individuals belonging to more than one stigmatized group may face heightened discrimination, shaped by the unique combination of social and cultural assumptions associated with their intersecting identities [32]. Intersectionality provides a valuable framework for examining the tensions between the lived experiences of people with multiple intersecting identities and the systems of oppression they face [33]. Understanding how various forms of discrimination intersect provides critical insights into human rights advocacy and the pursuit of enhanced social inclusion [34]. We drew on intersectionality to inform our methodological design as it enables the capturing of lived experiences and the complex social positions individuals occupy [35].

### Novelty of this study

Our study is novel because it addressed a critical gap in the literature. We explored recommendations from racially minoritized youth with disabilities for addressing ableism and other forms of discrimination. A recent review on the impact of race and ethnicity on school and work outcomes highlighted the limited focus on racially minoritized youth with disabilities, especially from a Canadian perspective [3]. Other recent reviews similarly emphasize the need for further research to untangle the complexity of how disability intersects with race and ethnicity [26]. It is crucial to explore the experiences and perspectives of racially minoritized youth with disabilities to help enhance their inclusion, access to services, and well-being [26]. Our research question was: what shifts to policy and practice are needed, including resources deployed and barriers removed, to ensure that youth with disabilities thrive in all their diversity?

## Methods

### Design and sample

Our objective was to explore recommendations made by racially minoritized youth with disabilities for addressing the discrimination and barriers they encounter. Using a qualitative descriptive design [36,37], we conducted in-depth individual interviews. We received ethics approval from our institutional research ethics board (REB-0533). We recruited participants using a purposive sample by sending mailed and emailed invitation letters through an internal *Connect2Research* database from a pediatric rehabilitation hospital. We recruited participants from a large urban, multicultural city, which is an optimal location given its status as one of the most culturally diverse cities in the world where 52% of the residents identify as members of visible racial and ethnic minority groups. We distributed flyers about our study at disability-related community organizations to invite people to participate. Interested participants reached out to the research team and a research co-ordinator screened them through a phone call or Zoom meeting. We applied the following inclusion criteria: (1) participants had to have a disability, defined as impairment in body function, structure, activity limitations and participation restrictions (World Health Organization definition) [38]; (2) be between 15 and 29 years of age based on Canada’s definition of youth [39]; (3) self-identify as belonging to a racially, minoritized, non-white, group that has experienced marginalization or discrimination based on racial identity [9]; and (4) be able to communicate in English independently or with the assistance of an interpreter or communication device and possess the cognitive capacity to participate. Youth who met the inclusion criteria read and provided informed written e-consent and documented via *REDCap* software. Parental signature was not required for this study. Participants received a $20 Canadian gift card as a token of appreciation as recommended by the REB. The interviewers and research team did not have any prior relationships or conflicts of interest with the participants.

Our sample involved 20 youth and young adults with a disability who identified as belonging to a racially minoritized group including: East Asian (11), South Asian (5), Middle Eastern (3), Southeast Asian (3), and multiracial (1). Participants ranged in age from 15 to 29 years (mean age 23 years) and had various types of physical and non-apparent disabilities including: autism; visual, hearing, speech, and cognitive impairments; mental health conditions (i.e., anxiety and depression); acquired brain injury; attention deficit hyperactivity disorder; obsessive–compulsive disorder; chronic pain; neurological disability; learning disability; epilepsy; complex post-traumatic stress disorder; Down syndrome; physical disabilities; cerebral palsy; and an undisclosed disability. Eleven youth reported having multiple disabilities. The sample involved 12 cisgender women, 4 cisgender men, 2 non-binary youth, 1 transgender man, and 1 gender fluid youth (see Table 1).

**Table 1:** Participant demographics.

ID #	Disability	Education Status	Employment Status	Age	Gender	Racial Category
1	Autism	In university	Part-time	24	Cisgender woman	Middle Eastern and South Asian
2	Visual and hearing impairments	Not in school	Part-time	29	Cisgender woman	East Asian
3	Speech impairment	Not in school	Unemployed	22	Cisgender woman	Middle Eastern and white
4	Anxiety and depression	Not in school	Part-time	26	Non-binary	East Asian
5	Acquired brain injury	In university	Full-time	25	Cisgender woman	East Asian
6	Hearing impairment, ADHD, depression, anxiety, OCD, autism, chronic pain, neurological disability*	Not in school (but in educational program)	Full-time	27	Gender fluid	East Asian and Southeast Asian
7	Depression and anxiety	Not in school (just graduated university)	Full-time	25	Non-binary	East Asian
8	Physical disability* and cognitive disability	In university	Part-time	22	Cisgender woman	Middle Eastern
9	Learning disability, ADHD, visual impairment	Not in school (just graduated university)	Unemployed	29	Cisgender man	South Asian
10	Epilepsy	In university	Part-time	22	Cisgender woman	Southeast Asian
11	Autism and complex PTSD	Not in school	Unemployed	25	Trans masculine, non-binary	East Asian
12	Autism and ADHD	In university	Part-time	23	Cisgender woman	Southeast Asian
13	Undisclosed disability*	In high school	Unemployed	18	Cisgender woman	East Asian and white
14	Physical disability*	In high school	Part-time	17	Cisgender woman	South Asian
15	Hearing loss and autism	In university	Unemployed	21	Cisgender man	East Asian
16	Down Syndrome	Not in school (just graduated high school)	Unemployed	17	Cisgender man	East Asian and white
17	Physical disability	Not in school	Full-time	28	Cisgender man	South Asian
18	Physical disability and cognitive differences	In university	Unemployed	18	Cisgender woman	East Asian
19	Physical disability, hearing loss, partial visual impairment, cognitive differences	In high school	Unemployed	15	Cisgender woman	East Asian and South Asian
20	Cerebral palsy	Not in school	Unemployed	27	Cisgender woman	Multiracial

*Disability type anonymized to protect participant confidentiality

### Data collection and analysis

Two cisgender racially minoritized women with academic backgrounds in sociology and rehabilitation sciences conducted the interviews. The semi-structured interview guide was informed by systematic reviews conducted by our team and an intersectionality framework [28]. We piloted the guide with two racially minoritized youth with disabilities prior to conducting all the interviews. Questions centred around recommendations to address discrimination such as ableism and racism, suggestions for removing or reducing barriers, required resources, and priorities for change. We collected demographic characteristics including age, gender, education status, employment status, type of disability, and race. We conducted interviews online through Zoom from November 1, 2023, to August 30, 2024, which lasted an average of 48 minutes. Interviews were audio-recorded and transcribed through Zoom, then verified for accuracy and de-identified. We applied an inductive thematic analysis guided by our research question [40]. Three authors, each bringing distinct vantage points from sociology, child disability, gender studies, along with lived experiences of minoritized identities such as disability, gender, and race analyzed the data. We acknowledge that our identities and social positions influence both the privileges we hold and the challenges we face encountering discrimination. We made a conscious effort to avoid making assumptions based on our own experiences and opinions.

We each read all the transcripts and independently developed initial codes, while noting patterns around addressing barriers and changes to policy and practice. The second author collated the codes and developed a codebook, which was reviewed, discussed and collectively agreed upon. We continued revising and organizing the codes until we reached consensus on the final codebook. The second author applied the codes to 50% of the transcripts using NVivo. Another team member then independently coded the same transcripts after which we calculated kappa scores to assess appropriate intercoder agreement [41]. Our team agreed upon the final codebook, and we discussed any discrepancies before re-coding all transcripts. After reviewing approximately 16 transcripts we noticed that no new codes were emerging in subsequent interviews and concluded that thematic and code saturation was reached [42]. We assessed the quality of the collected data and confirmed its suitability for an in-depth analysis [43]. We identified all relevant codes through discussion and agreed that the interview data provided a comprehensive understanding of potential solutions to address discrimination. We selected quotes to illustrate each theme and sub-theme [40].

We used the following approaches to enhance the trustworthiness of the findings. First, we applied a consistent interview guide for all the interviews. Second, the final coding framework was reached through team consensus and all decisions regarding coding and analysis were documented, enriching the credibility of the results [44]. Third, to better represent participants’ experiences, we drew on descriptive participant accounts in our analysis [44]. We reflected on how our positions and backgrounds, which included pediatrics, sociology and rehabilitation sciences, in addition to lived experience with disabilities and racially minoritized identities, may have influenced the development of the themes [44]. Finally, the Consolidated Criteria for Reporting Qualitative Research guidelines were followed [45].

## Results

Four main themes were identified including: (1) addressing barriers in healthcare, education, employment, and the legal system (i.e., understanding multiple minoritized identities, training and education); (2) community, social supports and resources (i.e.,i.e., mentorship and support groups, support with accessing relevant resources and services); (3) advocacy (i.e., self-advocacy, representation of multiple minoritized identities); and (4) inclusive policies and youth involvement (see Table 2).

**Table 2:** Exemplar participant quotes.

Theme 1. Addressing barriers in healthcare, education, employment, and the legal system	
**Understanding multiple minoritized identities**	“Some services would be great for people with physical disabilities, but they’re not really aware of how gender or race may impact a person’s experiences and their physical disability…There needs to be an intersectional lens from any organization that is providing services or interacting with people with disabilities…A person’s needs and wants, or how they interact with society is obviously not based off only one element of the identity.” (#17, South Asian cisgender man) “In regard to being racially marginalized, I don’t see it as being completely separate factors to being disabled… it’s just an additional compounding factor. I don’t see it as being exclusive. I feel like it goes down to the prejudices that people have, and the predisposed assumptions that people form about you because of what they could see about you.” (#8, Middle Eastern cisgender woman)
**Training and education**	“The most important thing is education…I learned about discrimination…They only really talked about race discrimination, or even if it was disability discrimination, it was the most stereotypical type of disability discrimination. It would usually be a kid in a wheelchair, and that’s it. But disabilities range so much more than just a wheelchair.” (#18, East Asian cisgender woman) “The stigma, when you look at ableism, is very negative. So again, the education for the people you put in there…they should be actually trained ﬁrst.” (#16, East Asian cisgender man)
**Theme 2. Community, social supports and resources**	
**Mentorship and support groups**	“It’s the collective understanding of those with disabilities in terms of wanting to help each other because everyone’s been through some sort of experience where they felt excluded or felt like their clinicians could have done more.” (#5, East Asian cisgender woman) “If we could tell neurologists or epileptologists, instead of just focusing on the medications and the side effects, which don’t get me wrong is important, ask about the social life. Are there any support groups that you could maybe inform them about? Maybe give them a brochure?” (#10, Southeast Asian cisgender woman)
**Support with accessing relevant resources and services**	“Healthcare services have trouble with being culturally sensitive...There might be gaps in knowledge, and it’s up to the service providers willingness to learn…about being non-discriminatory, about histories, about different cultures and different lifestyles. A lack of cultural sensitivity can make it so that the quality of care is lower…I think that folks with more marginalized identities and different intersections factor in…how difficult it might be to find somebody that is willing to help.” (#4, East Asian, non-binary) “I find that a lot of racialized and particularly if you’re disabled and you’re a visible minority. That puts you in this unique position, where you’re going to receive kind of twice the discrimination, as somebody who is say, able-bodied or does not appear disabled…I feel there could be a lot of improvements…if a disabled, visible minority is seeking help, there’s just way too many access barriers for that.” (#11, East Asian transman)
**Theme 3. Advocacy**	
**Self-Advocacy**	“They should be treated just as everyone else. Just because they’re marginalized doesn’t mean they need to be looked down on just because of how they appear…Everyone knows themselves, and they should have the right to speak for themselves.” (#14, South Asian cisgender woman) “If you’re thinking about the healthcare space, having people that maybe more of their role is dedicated to being an advocate for these youth with disabilities for people with disabilities. And always having that be an important piece of the organization, important piece of the services.” (#4, East Asian non-binary)
**Representation of multiple minoritized identities**	“Having people who speak the language. That’s the ﬁrst. Information has to be in their language ﬁrst and foremost. So, it cuts another layer of that…It doesn’t even have to be like health professionals. It could be education, or just to see people that are of the same. The visual aspect needs to kind of be there, right?” (#16, East Asian cisgender man) “We should be showing people that disability is not just a physical trait, but also a mental trait. Normal people who can walk and talk may be struggling with their own disabilities. And we should be able to show people that even people with disabilities, physical or mental, can do the same things as an able-bodied person.” (#19, East Asian cisgender woman)
**Theme 4. Inclusive policies and youth involvement**	
	“The first step would be to understand what type of ableism and or discrimination actually exists. So, the only way to do that is to empower the lived experience of youth with disabilities, and hear from them…It would be important to have some sort of consultation…that seeks to understand what type of barriers that youth are actually facing today and work to build policy around that…giving them an equal footing to access whatever it is that they need in their daily lives.” (#17, South Asian cisgender man) “Even though people say they have AODA [Accessibility for Ontarians with Disabilities Act], or inclusive hiring policies…if people say they have a disability on the application…they’re less likely to be hired. That should be removed. People should have equal chances of getting hired, and maybe that would involve just not asking people to disclose at all.” (#12, Southeast Asian cisgender woman)

### Theme 1. Addressing barriers in healthcare, education, employment, and the legal system

Racially minoritized youth with disabilities emphasized the need to address barriers in healthcare, education, employment, and the legal system. Participants reported experiencing discrimination, struggling to access necessary services, and receiving inadequate support from service providers and employers. They proposed several strategies to address these challenges, such as increasing understanding of how intersecting minoritized identities shape experiences of inclusion and discrimination, and enhancing training on disability awareness, anti-discrimination and multiple minoritized identities.

*Understanding multiple minoritized identities.* Fifteen participants discussed the importance of considering racial and ethnic identities alongside their disability and other minoritized identities. Stereotypes and prejudice regarding their disability often created barriers for youth. Often, the challenges of racially minoritized youth with disabilities were exacerbated due to additional discrimination towards their racial and ethnic identities, which led to poor access and lowered quality of essential services. A Southeast Asian cisgender woman highlighted “for racialized people, it’s even more challenging because there’s that extra barrier and extra things to be afraid of” (#12).

Participants suggested that service providers, employers, and organizations should develop a better understanding of multiple minoritized identities, and the many forms of discrimination they may encounter. By learning about the barriers that multiply minoritized participants encounter, individuals and organizations can implement anti-discriminatory practices and policies to facilitate greater access to resources and better quality of services. An East Asian non-binary participant noted, services should consider how their ethnicity and disability work in tandem:

“It’s not a holistic view without it, because as much as it’s important to give each piece of identity its own time…you also have to look at the overall picture because a person isn’t just one thing you know…You don’t go through the world and be like, today I am just a Chinese person…Maybe I go into a Chinese community, and I’m still talking about how does my disability factor into it? It’s just back and forth different pieces, interweaving with each other the very idea of it being intersectional…you can’t separate those things.” (#4)

*Training and education.* Nineteen participants suggested that service providers and organizations engage in further training and education to develop a nuanced understanding of multiple minoritized identities and anti-discrimination. Participants recommended an approach to training and education that considers how their multiple minoritized identities contributes to their diverse experiences, perhaps through increased workshops and resources. They felt greater education and training on these topics would help service providers deliver more inclusive services and reduce multiple forms of discrimination. Participants suggested that service providers make time to have more discussions around “what EDI [equity, diversity, and inclusion] is or what IDEA [inclusion, diversity, equity, and accessibility] is” to ensure that they understand key terms, while “having a chance to sort of share what they are experiencing” (#5, East Asian cisgender woman).

Participants recommended that service providers learn more about providing culturally sensitive care, and how culture influences understandings of disability. For instance, some cultures may view disability negatively, which can lead to ableism. Participants sometimes “have to pick up the pieces because…parents would think that [children with disabilities are] possessed by a demon or a witch…those type of cultural myths are still very much alive” (#10, Southeast Asian cisgender woman). Rather than the onus being on youth to challenge cultural views of people with disabilities and ableism, service providers should be more adequately prepared to do so by providing culturally sensitive care. Participants explained that people with disabilities should help develop training and education for service providers and employers, and that training should be regularly updated to ensure that the information is current and accurate.

Participants indicated that increased training could enhance safety and care for racially minoritized youth with disabilities. Participants reported being aware that “healthcare providers or teachers will have biases…and maybe put racialized groups of students who are disabled too, they’d be put at the back of the line when it comes to stuff like getting healthcare” (#19, East Asian cisgender woman). For participants, equal and non-discriminatory access to care was important, and “a major safety concern for just peace of mind…for anybody who has had an identity that is often treated unkindly by society at large” (#11, East Asian transman). Participants suggested that service providers should confront personal biases or stereotypes about racially minoritized youth with disabilities to facilitate greater trust with their clients, which may lead to improved healthcare access and outcomes:

“Depending on how the healthcare practitioner interacts with folks, it really shows you if you can build that trust with that person and I think if we instill these ideas of representation, inclusion, then that really keeps the people who are doing this discrimination to do the work themselves as opposed to having the folks that are victimized be the ones having to explain ourselves.” (#20, multiracial cisgender woman)

### Theme 2. Community, social supports and resources

Participants used community and social resources to cope with encounters of discrimination. They turned to support groups and mentors who understood their multiple minoritized identities. Most participants described a need for more opportunities for mentorship and support groups that are disability-focused and that highlight their racial and ethnic identities. Such opportunities could enhance youth belonging and inclusion. Participants faced compounded barriers based when they navigated relevant resources or services due to their disability, race, and ethnicity. They suggested that organizations provide greater support for accessing resources and services so that youth can receive the assistance they need.

#### Mentorship and support groups.

Twelve racially minoritized participants explained how mentorship opportunities and support groups helped them cope with barriers and discrimination. Participants with disabilities appreciated sharing their experiences with others who faced similar challenges based on their minoritized identities among mentors and in support groups. Moreover, mentors and group members encouraged and comforted them by their willingness to help one another. Participants encouraged other young people with disabilities to network and find others with similar experiences: “3 to 5, 10, years ahead of where you want to be and…make sure you can get good guidance and mentorship because there are barriers and learning how to navigate them is important” (#9, South Asian cisgender man). An East Asian non-binary participant discussed the importance of having mentors who understand the experience of having multiple minoritized identities: “BIPOC [Black, Indigenous, and People of Color] people who come from immigrant families…having people who also come from a similar culture to mentor you or be a part of similar support groups would be helpful” (#7).

Engagement in relevant support groups enhanced youth’s social inclusion. Support groups often provided safe spaces for minoritized participants who lacked a sense of security in other environments due to multiple forms of discrimination. Participants suggested that they develop more specific support groups that highlight their disability, race, and ethnicity. An East and South Asian cisgender woman suggested “youth can find people that relate to them when it comes to their situations and racism or sexism, and they can share their experiences together, so they don’t have to feel alone” (#19). Given the value of mentorship and support groups, participants advocated for more funding to enable organizations to provide relevant services that are crucial for supporting multiply minoritized people with disabilities. Participants described how community organizations often have limited funding and suggested removing restrictions on how organizations can use their financial support.

### Support with accessing relevant resources and services

Thirteen participants encouraged others to access relevant resources and services. Racially minoritized participants described how discrimination against their disability, race, and ethnicity creates multiple barriers when they try to access services. They explained that service providers and organizations should employ better strategies to help youth who may encounter a combination of ableism and racism when accessing services, as recommended by participants. This includes redistributing resources to multiply minoritized youth, using various methods to communicate information, and connecting youth to disability and culturally specific organizations.

Racially minoritized participants with disabilities sometimes faced greater discrimination or received limited information about essential services. This prevented participants from accessing the support they need. For instance, a participant recalled that if their “peers were not a person of color, they’re shown more empathy, and they’re given more support compared to me and they had always just been given pamphlets to just take home and read it over myself, kind of thing, very dismissive in a way” (#1, Middle Eastern/South Asian cisgender woman).

Participants sometimes avoided using certain services because they feared experiencing multiple forms of discrimination. Further issues often arose, such as health complications. As an example, a multiracial cisgender woman avoided accessing healthcare services after she experienced severe abdominal pain. She feared that “as someone that is conceived to be a Black woman…and because of my own internalized trauma, I was telling myself that I don’t have to go to the hospital…Nobody’s gonna listen to me if I go to the hospital” (#20). She further recommended that we provide more support for racially minoritized youth with disabilities who avoid asking for assistance.

Some participants were unaware of the services available to them. They proposed better guidance on relevant resources and more services for racially minoritized youth with disabilities. They also emphasized the need to examine the location of available resources. Some services might exist in one city, but not others. This situation leaves certain individuals without the support they need. Service providers should consider multiple minoritized identities when delivering services and resources. Participants suggested distributing resources to those who need them the most. In some cases, these are:

“People who have been forcibly disabled in some way, which is most often gender and racial minorities. We should be distributing resources in such a way where the people who need the most care get it first, and that is the case here, where those who have the intersection of being disabled, of being racialized, of being a gender minority should definitely get that care first and should have that specific care.” (#6, Southeast Asian, gender fluid)

Participants advocated for various ways to communicate information, especially for hard-to-reach populations, such as newcomers or individuals from low-income backgrounds. A South Asian cisgender man suggested: “you need to have a multi-faceted communication approach…For a marginalized community, you might need to actually go to that community center to interact with them and sort of hand out physical ﬂyers about the type of programs that are available to them” (#17).

Participants also encouraged service providers to connect their clients with resources for minoritized groups, such as disability or culturally specific organizations. Participants explained that some racially minoritized groups, such as newcomers with disabilities or individuals who encounter English language barriers, need additional support to access resource and services. Participants suggested that services provide more language translators to ensure that people with disabilities understand their healthcare options or information about disability rights. For instance, an East Asian cisgender woman described the struggle involved in finding support: “When we were in the ER, there were no nurses who could speak a language that he could understand. So maybe we could have more translators” (#18).

### Theme 3. Advocacy

Racially minoritized participants described the importance of advocacy to reduce discrimination and raise awareness of their challenges encountered. Participants encouraged more opportunities to discuss their experience of multiple forms of discrimination with service providers, and strategies for addressing their challenges. Many participants discussed the need for greater representation of multiple minoritized identities. Multiple minoritized identities enabled participants to engage with others who had similar experiences, which could facilitate their sense of inclusion and belonging. Representation can highlight the achievements of multiply minoritized people with disabilities.

#### Self-Advocacy.

Six participants mentioned the importance of self-advocacy, including raising their concerns to others, knowing their rights, and finding solutions to their challenges. Participants faced insufficient opportunities to express themselves, especially about the discrimination they experienced. They recognized the difficulty racially minoritized people with disabilities face when speaking out. A participant noted: “When you compound diﬀerent minority factors or diﬀerent discriminatory factors…it just becomes harder, but by creating the ground, it still opens up some capacity” (#8, Middle Eastern cisgender woman). Participants explained how healthcare providers have ignored or dismissed concerns raised by racially minoritized individuals with disabilities. Participants suggested that service providers should give more time and opportunities with service providers to discuss their experiences of discrimination. These conversations could also help build trust and rapport between service providers and youth, leading to better care. A participant expressed this idea:

“Having more opportunities for the clinician and family to talk about these things more openly, because, again, this is all structural limits, in terms of appointment times are only this amount of time. But even having each clinician…checking in like, is there anything else you want to talk about? Just asking that question about sharing an experience of discrimination or social exclusion that otherwise wouldn’t have been discussed…being able to ask about that is really important for building the opportunity for discussion and sharing.” (#5, East Asian cisgender woman)

Participants suggested more advocacy from service providers and reported that these providers often failed to take the time to properly address their concerns, which led to confusion and a lack of patience in healthcare spaces. Participants recommended that healthcare organizations should hire staff dedicated to anti-discriminatory initiatives. A multiracial cisgender woman suggested: “whether it’s educational, employment wise or healthcare, it would be amazing to see one day someone or multiple people being in these organizations, these systems, for the sole purpose of upholding anti-racist and anti-ableist practices” (#20). Staff or service providers could advocate for multiply minoritized youth with disabilities to help reduce the discrimination they face and enhance their well-being.

#### Representation of multiple minoritized identities.

Eleven participants observed that education, healthcare, and employment lacked disability, racial or ethnic representation. Participants felt ignored and misunderstood without representation of their minoritized identities. Participants advocated for greater representation of their multiple minoritized identities, which can help youth feel more included and more comfortable, potentially leading to better well-being. These individuals would bring “in their own lived experience and that way, they’re better able to understand my needs, compared to someone who doesn’t quite look like me” (#1, Middle Eastern cisgender woman). Participants further clarified that minoritized staff should also understand the challenges that racially minoritized youth with disabilities face. Employers should not hire them just to simply fulfill diversity quotas or for tokenistic reasons:

“It would help to have people on the team who are also racialized and pretty much the whole diversity thing. But really diverse people who understand disability and not just trying to fill out a quota or anything. It’s just people who have lived experience, who are committed to the cause. I think having that presence is really important.” (#12, Southeast Asian cisgender woman)

Participants suggested that alternative forms of care, such as including cultural practices and diets, could improve inclusion and reduce discrimination in health care. For example, a participant remembered staying at a hospital and struggling to eat Western style food. She explained “I didn’t really like the food, and it didn’t help my care that much…if hospitals could make food for a Western culture, it would be nice if they could also make one for an Eastern culture” (#18, East Asian cisgender woman).

Participants observed that some spaces designed for disability inclusion fail to embrace other minoritized identities, such as racial or ethnic identities, which can result in discrimination. An East and Southeast Asian gender fluid participant recalled being looked at with disgust by “everyone who is white” while having a conversation with disability activists, who later “changed the topic and left me out of the conversation. And of course, I don’t know exactly what it was that made them stop, but it made me feel very excluded, and…it was the one thing that made me realize I was the only person of color at this table” (#6). Participants suggested that embracing multiple minoritized identities in all spaces is crucial to avoid similar discriminatory and isolating experiences.

### Theme 4. Inclusive policies and youth involvement

Nine participants expressed a critical need for inclusive policies that acknowledge and challenge discrimination against multiple minoritized identities. Inclusive policies can lead to better support, greater inclusion, and reduced discrimination. Participants further suggested that the policy and decision-makers should incorporate their lived experiences and prioritize conversations about anti-ableism and anti-racism. This may be achieved by the participation of multiply minoritized youth in policymaking, which could also help enhance their advocacy skills.

Participants encouraged policy and decision-makers to develop initiatives that enable people with disabilities to feel valued and included. Inclusive policies should consider how individuals’ complex and overlapping identities can inform their needs. For example, a participant commented, “any policy or any practices that are implemented need to be very intersectional…Not only is it youth with disabilities…But there’s so many parts of a person’s identity that affect how they may be treated, or what sort of services or needs that they have” (#4, East Asian, non-binary). Participants observed that some existing policies overlooked how their disability, race, and ethnicity can impact their experiences. For instance, participants who attended post-secondary institutions faced challenges in obtaining culturally inclusive academic accommodations. An East and Southeast Asian gender fluid participant described receiving accommodations “based off of what a white man would need and obviously it wasn’t helpful to me” and instead suggested that “policies could be potentially and specifically geared towards bolstering the lives of those who are gender minorities, racialized minorities, because all of these issues overlap for sure” (#6).

Participants discussed their involvement and more opportunities in policymaking to help inform inclusive policies and services. They also emphasized the importance of policymakers listening to the lived experiences of youth with disabilities, as they are the experts on their needs. A multiracial cisgender woman explained, “to prevent discrimination and racism and ableism, these systems have to integrate and include people with disabilities at the table from volunteer work to client representation, to even working with the leadership teams so its reﬂected in policy and vice versa” (#20).

Including multiply minoritized individuals with disabilities in policymaking should also involve “more open discussion about race, disability, and other identities as there are not too many regular discussions about equity, diversity and inclusion” (#5, East Asian cisgender woman). Policy and decision makers can create opportunities for youth involvement, such as advisory councils, workshops, or roundtable discussions. Such opportunities could help to empower youth to advocate for their needs and facilitate social inclusion by feeling valued.

## Discussion

This study explored recommendations from racially minoritized youth with disabilities for reducing barriers and multiple forms of discrimination. Our study introduces novel insights by examining racially minoritized youth with disabilities in Canada using an intersectional framework to consider disability alongside race and ethnicity, an area where existing research remains limited [3,26]. Our findings demonstrate that multiply marginalized youth faced various barriers in healthcare, education, employment, and the legal system. Several changes to policies and practices that could facilitate greater inclusion and reduce discrimination were suggested by youth. The suggested changes are important to consider, as previous research shows how a lack of accessibility and inclusion can be detrimental to the health and social outcomes of youth with disabilities [46].

Most participants in our study proposed that service providers or employers should engage in more training to support people with disabilities, which aligns with previous research on the topic [47–49]. Specifically, individuals should learn more about how youth with disabilities’ multiple minoritized identities impact experiences of inclusion and discrimination [47,50]. Organizations should offer workshops and resources that highlight examples of discrimination against multiply minoritized people with disabilities. Organizations should provide strategies for mitigating discriminatory situations [47]. Ongoing training and education should occur throughout the various stages of professional practice [50].

Service providers should engage in training to explore how they might be oppressing others in daily interactions [48]. Individuals working with youth with disabilities should receive the resources they need to provide culturally sensitive care [49]. Training and education should implement a collaborative approach involving youth with disabilities, service providers, and employers [49]. Previous research shows that employing people with disabilities as instructors could effectively increase knowledge and disability awareness [51,52].

Our findings align with past research demonstrating that community and social support are key coping mechanisms for dealing with discrimination [53,54]. Our study found that trusted individuals provided support and safe and inclusive spaces for youth. Participants often engaged in support groups with people who shared similar minoritized identities as themselves. These individuals and groups helped youth by providing youth with information, guidance and companionship. Participants suggested a need for increased opportunities for mentorship and specific support groups that focus on disability, race, and ethnicity.

Our research highlights the strong need for individuals to have psychologically safe spaces in which they feel comfortable with interpersonal risk-taking, such as disclosing their identity, and contributing to ideas and actions within a shared group [55,56]. Researchers highlight the necessity to create psychologically safe spaces to reduce bullying, harassment, and conflict within groups and organizations [55]. Psychologically safe environments involve the following conditions: elicit a feeling of inclusion, provide safe learning experiences, provide an equitable opportunity to contribute, and support challenging the status quo while promoting curiosity [57,58]. These factors enable people to see the strengths of others, rather than focusing on differences [57].

Our study found that youth encountered barriers when they tried to access relevant resources and services. Participants recommended that organizations provide greater support with accessing essential services and resources through redistributing resources, finding multiple ways to communicate information, and connecting youth to disability and culturally specific organizations. Previous researchers demonstrate the benefits of considering disability alongside race and ethnicity when helping youth access relevant resources and programs, such as enhanced social and psychological well-being for youth and their families [59].

Our results align with previous research that shows advocacy may help youth cope with discrimination at both the individual and socio-structural levels [11]. Our participants encouraged more opportunities for voicing their concerns and learning about their rights, especially regarding experiences of discrimination. Representation of multiple minoritized identities is key to alleviating barriers and discrimination [60,61]. People with disabilities currently lack representation across various settings. To illustrate, researchers found that images of individuals with disabilities only appear in 29% of educational textbooks, demonstrating a lack of inclusion in education [61]. Healthcare education programs and professions often exclude and underrepresent people with disabilities due to discriminatory attitudes or practices [13,62]. Our participants suggested that organizations should hire more racially minoritized individuals with disabilities in healthcare, education, and employment. This hiring could increase a sense of inclusion and awareness of multiple minoritized identities.

To put these suggestions into place, participants recommended more inclusive policies that consider the complicated nature of multiple identity dimensions and intertwined systems of oppression [33,63]. Youth with disabilities should collaborate in creating inclusive policies, leading conversations about anti-ableism and anti-racism, and offering recommendations grounded in their lived experiences [64]. Workshops, roundtable discussions, or youth advisory councils could enable better support, more inclusion and less discrimination by including youth with disabilities in decision making and policy development [64]. Previous research shows that youth participation and facilitation of advisory meetings led to greater empowerment and resulted in non-discriminatory and equitable policies [64]. At the same time, policymakers should welcome youth recommendations and proposed changes [64].

Many participants in our study described using individual-level strategies to challenge discriminatory practices or attitudes. However, it is crucial to emphasize that society and organizations should take responsibility for addressing systemic barriers and mitigating discrimination [48]. Further research is required at multiple levels (e.g., individual, institutional and societal levels) to combat negative attitudes and behaviors towards racially minoritized people with disabilities through anti-discrimination initiatives [65,66]. Some researchers argue that anti-discrimination initiatives need to change the power that underpins discrimination to succeed [65].

## Implications

Our findings have important implications for services and policies. First, service providers, employers, and other individuals working with youth with disabilities must urgently pursue training and education on disability and other minoritized identities, including racial and ethnic identities. Understanding how multiple minoritized identities impact the experiences of people with disabilities can help in developing strategies to challenge discrimination. Furthermore, service providers should understand and apply principles of culturally competent care. Service providers, employers, and individuals with disabilities should collaborate to implement training that will have positive results. Second, we need to provide further community and social support to youth with disabilities. These supports can serve as coping mechanisms for youth who face discrimination due to their disability and other minoritized identities. Organizations providing essential social support to youth with disabilities should receive more funding. Service providers and clinicians should connect youth to disability and culturally specific resources and consider developing a centralized hub listing available supports. Third, society and organizations should provide individuals with disabilities with more opportunities to voice their concerns and advocate for their needs, particularly regarding the discrimination they encounter. Education, healthcare, and employment systems should include greater representation of multiple minoritized identities. Fourth, policy and decision makers should include the lived experiences of multiple minoritized youth with disabilities and prioritize conversations about anti-ableism and anti-racism. Youth with disabilities can contribute to inclusive policies by participating in advisory councils, roundtable discussions or workshops. Youth involvement in policy development can enhance their advocacy and decision-making skills, develop social connections, and improve their confidence, and ultimately lead to better outcomes for their well-being and future success.

## Limitations and future directions

It is important to consider the limitations of our study. First, we recognize that people often perceive and treat people with disabilities differently across cultures, and it can be difficult to discern the extent of their experience of discrimination. Additionally, our team recruited the sample from one location that may not represent youth from other geographic locations. We also had an unintentional over-representation of cisgender women in our sample. These limitations limit the generalizability of the findings. Future studies should consider larger samples and consider focusing on specific types of disabilities, and racially and gender minoritized groups for a more in-depth exploration of discrimination’s impact. Future research should examine how youth experience racism and ableism, and the situations and contexts in which these occur. In addition, exploring other aspects of minoritized identities, such as sex/gender, social class and geographic location may be fruitful. Finally, researchers could explore how to develop and maintain inclusive and safe spaces for people with multiple minoritized identities.

## Conclusions

Using a qualitative approach, our study explored the changes that policy and decision makers need to implement to reduce barriers and discrimination faced by racially minoritized youth with disabilities. Our findings showed that youth encounter many forms of discrimination based on their multiple minoritized identities, including ableism and racism. Racially minoritized youth faced barriers in healthcare, employment, education, and the legal system. Our results demonstrated that further awareness is necessary regarding how multiple minoritized identities can impact experiences through training and education. We need to increase mentorship opportunities and support groups and enhanced support for accessing relevant resources and services. We must provide more opportunities for self-advocacy and greater representation of multiple minoritized identities in decision-making. We need to promote more inclusive policy making in collaboration with youth. The proposed changes to policy and practice will help youth access the resources they need, enabling them to enhance their quality of life and wellbeing.
